# The neurotransmitter dopamine modulates vascular permeability in the endothelium

**DOI:** 10.1186/1750-2187-3-14

**Published:** 2008-07-28

**Authors:** Resham Bhattacharya, Sutapa Sinha, Su-Ping Yang, Chittaranjan Patra, Shamit Dutta, Enfeng Wang, Debabrata Mukhopadhyay

**Affiliations:** 1 Department of Biochemistry and Molecular Biology, Mayo Clinic College of Medicine, Rochester, MN, USA; 2 Department of Pathology, Massachusetts General Hospital, Boston, MA, USA

## Abstract

**Background:**

Vascular permeability factor/Vascular endothelial growth factor (VPF/VEGF), a multifunctional cytokine, is a potent inducer of vascular permeability, an important early step in angiogenesis. It is known that the neurotransmitter dopamine can inhibit VPF/VEGF mediated angiogenesis, in particular microvascular permeability, but the effectors of this action remain unclear.

**Results:**

Here, we define the signaling pathway modulated by dopamine that inhibits VPF/VEGF induced vascular permeability in endothelial cells. Signals from VPF/VEGF lead to changes in the phosphorylation of tight junction protein zonula occludens (ZO-1) and adherens junction proteins like VE-cadherin and associated catenins, thus weakening endothelial cell-cell adhesion and increasing vascular permeability. We found VEGF receptor-2 (VEGFR-2) to be part of a multi-protein complex involving ZO-1, VE-cadherin and β-catenin. VPF/VEGF induced phosphorylations of VE-cadherin, β-catenin and ZO-1 were inhibited by dopamine treatment. Association of occludin with ZO-1 and ZO-1 with VE-cadherin were significantly inhibited by dopamine in VEGF treated cells. Furthermore, we identified Src as an important target for dopamine-mediated inhibition of VPF/VEGF induced permeability.

**Conclusion:**

Taken together, our results provide molecular insights of dopamine function in the vascular endothelium and suggest a central role of Src in regulating key molecules that control vascular permeability.

## Background

Vascular permeability factor/vascular endothelial growth factor (VPF/VEGF) is a multifunctional cytokine that plays a critical role in angiogenesis [1-3]. It is a pleiotropic growth factor that mediates multiple functions via stimulation of its cognate receptors on endothelial cells [1-3]. VPF/VEGF was originally described as a tumor-secreted protein that renders venules and small veins hyperpermeable to circulating macromolecules and was, therefore, initially termed vascular permeability factor [1-5]. In fact, VPF/VEGF is one of the most potent inducers of vascular permeability known. It is 50,000-fold more potent than histamine [1,3-6]. This ability to enhance microvascular permeability remains one of the most important properties of VPF/VEGF, especially with regard to the hyperpermeability of tumor vessels that is attributable to tumor cell expression of VPF/VEGF [1,3-6]. Furthermore, it has been suggested that the increase in permeability results in the leakage of several plasma proteins, including fibrinogen and other clotting proteins. This can lead to the deposition of fibrin in the extravascular space, which subsequently retards the clearance of edema fluid and transforms the normally antiangiogenic stroma of normal tissues into a proangiogenic environment [1,3-5]. VPF/VEGF increases permeability in a variety of vascular beds – including those of the skin, peritoneal wall, mesentery, and diaphragm – and can lead to pathologic conditions such as malignant ascites [3,7] and malignant pleural effusions [3,8]. In fact, there is evidence that inhibition of VPF/VEGF can lead to decreased formation of pleural effusions [3,8] and that antibodies directed against VPF/VEGF or VEGFR-2 can lead to a decrease in tumor vessel permeability and ascites formation [1,3,8-10].

Vascular permeability, as induced by VPF/VEGF, is due to its action on the endothelium. Endothelial cell-to-cell junctions are complex structures formed by different adhesive molecules [11,12]. Endothelial cells have tight junctions (TJ) and adherens junctions (AJ). AJ are formed by transmembrane proteins that belong to the cadherin superfamily such as vascular endothelial VE-cadherin [11,13]. These proteins are linked inside the cells to β-catenin and plakoglobin that in turn, through the binding to α-catenin, promote anchorage to the actin cytoskeleton and are required for full control of junctional permeability [14]. TJ, in contrast, are formed by three different types of transmembrane proteins: occludin [15], claudins [16], and junctional adhesion molecule (JAM) [17]. Inside the cells, several cytoskeletal signaling molecules are concentrated in the TJ area, such as ZO-1, cingulin, and 7H6 [18,19].

We have recently demonstrated that dopamine significantly and specifically inhibits VPF/VEGF induced vascular permeability in the endothelial cells *in vivo *[20-23]. However, the effectors by which dopamine mediates this action is unclear [20-23]. VPF/VEGF induced increase in vascular permeability is either through disruption or change in localization of ZO-1 and VE-cadherin between the endothelial cells [6,24-27]. Reports also show that quiescent blood vessels contain a complex involving VEGFR-2, VE-cadherin and β-catenin that are transiently disrupted by VPF/VEGF injection. Blockade of Src prevents disassociation of this complex and inhibits edema accumulation [28,29]. Accordingly, we investigated whether dopamine mediated inhibition of VPF/VEGF induced vascular permeability is a result of its antagonism of VPF/VEGF induced disruption of VEGFR-2, ZO-1, β-catenin and VE-cadherin complex in the endothelial cells. We also investigated if dopamine mediated modulation of TJ and AJ proteins in the vascular endothelium in response to VPF/VEGF were Src dependent.

## Results

### Dopamine inhibits VPF/VEGF mediated in vitro permeability

**Figure 1:**
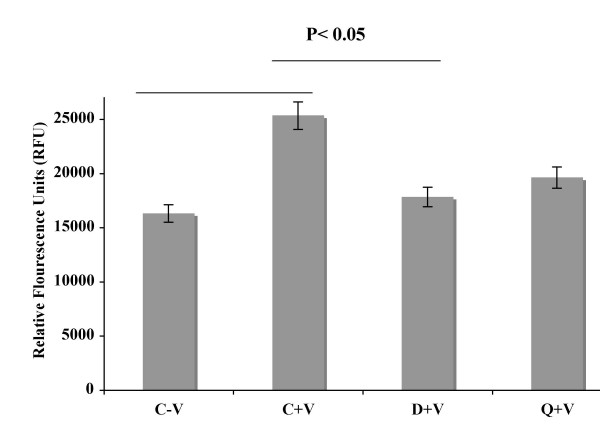
**Dopamine inhibits VEGF induced *in vitro *permeability in HUVEC**. Permeability is represented by relative fluorescence units as measured by a flux of FITC-Dextran across monolayer of HUVEC cultures in collagen-coated transwells. In the figure C-V is the control, HUVEC without any VPF/VEGF or dopamine treatment, C+V is the HUVEC treated with only VPF/VEGF (10 ng/ml) for 90 min, D+V is the HUVEC pre-treated with 10 μM dopamine for 15 min and then treated with VPF/VEGF (10 ng/ml) for 90 min and Q+V is the HUVEC pretreated with 10 μM quinpirole for 15 min and then treated with VPF/VEGF (10 ng/ml) for 90 min. Experiments were repeated three times in triplicate.

VPF/VEGF induced permeability significantly increased in cultured HUVEC compared to the untreated cells. However, permeability significantly decreased when HUVEC were pre-treated with 10 uM dopamine or the specific dopamine D_2 _receptor agonist quinpirole before administering VPF/VEGF (Figure 1). These results are consistent with our previous *in vivo *findings where we demonstrated that dopamine significantly and specifically inhibited VPF/VEGF induced vascular permeability in the endothelial cells [20-23]. Here we have focused on the possible mechanism by which dopamine mediates this inhibition.

### Dopamine modulates association of VEGFR-2 with Src

**Figure 2:**
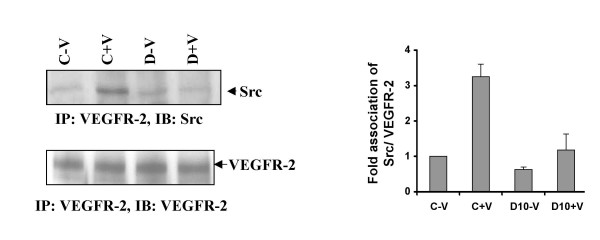
**Dopamine treatment alters VEGF/VPF induced VEGR-2 and c-Src association in HUVEC**. HUVEC extracts were immunoprecipitated with VEGFR-2 antibody and immunoblotted with Src and VEGFR-2 antibody. In the figure C-V is the control, HUVEC without any VPF/VEGF or dopamine treatment, C+V is the HUVEC treated with only VPF/VEGF (10 ng/ml) for 15 min, D-V is the HUVEC treated with 10 μM dopamine for 15 min and D+V is the HUVEC pretreated with 10 μM dopamine for 15 min and then treated with VPF/VEGF (10 ng/ml) for 15 min. Q-V is the HUVEC treated with 10 μM quinpirole for 15 min and Q+V is the HUVEC pretreated with 10 μM quinpirole for 15 min and then treated with VPF/VEGF (10 ng/ml) for 15 min. The figures are representative of three separate experiments with similar results.

We demonstrated by immunoprecipitation in HUVEC that Src remained constitutively associated with VEGFR-2, and this association increased with VPF/VEGF treatment. However pre-treatment with dopamine significantly inhibited this association (Figure 2).

### Activation of Src is dependent on tyrosine phosphorylation of VEGFR-2

**Figure 3:**
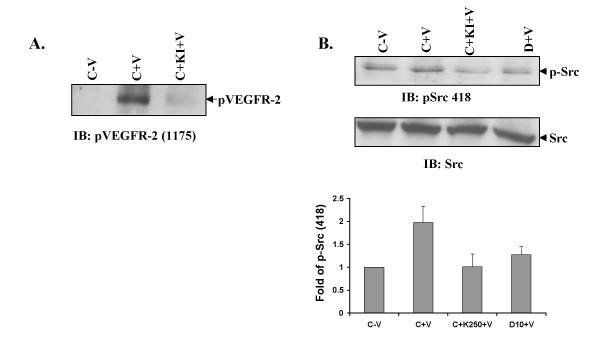
**VPF/VEGF induced VEGFR-2 phosphorylation is required for Src phosphorylation**. (A) Kinase inhibitor (250 nM) inhibits VPF/VEGF induced VEGFR-2 Y1175 phosphorylatin. In the figure C-V is control, HUVEC without any VPF/VEGF or dopamine treatment, C+V is the HUVEC treated with only VEGF (10 ng/ml) for 15 min, C+KI+V is the HUVEC pretreated with kinase inhibitor (KINASE IV) for 30 min and treated with VPF/VEGF (10 ng/ml) for another 15 min (B) Pre-treatment of HUVEC with kinase inhibitor inhibits VEGF induced Src Y418 phosphorylation. In the figure C-V is control, HUVEC without any VPF/VEGF or dopamine treatment, C+V is the HUVEC treated with only VPF/VEGF (10 ng/ml) for 15 min, C+KI+V is the HUVEC pre-treated with kinase inhibitor for 30 min and treated with VPF/VEGF (10 ng/ml) for another 15 min and D+V is the HUVEC pretreated with 10 μM dopamine for 15 min and then treated with VPF/VEGF (10 ng/ml) for 15 min. The figures are representative of three separate experiments with similar results.

Next we wanted to determine if activation of Src was dependent on phosphorylation of VEGFR-2. At first we confirmed efficacy of the kinase inhibitor (KINASE IV) by treating HUVEC with the inhibitor with or without VPF/VEGF and determined that phosphorylation of Y1175 on VEGFR-2 was significantly decreased (Figure 3A), subsequently total tyrosine phosphorylation of VEGFR-2 was also decreased (data not shown). Next HUVEC were pre-treated with dopamine or kinase inhibitor with or without VPF/VEGF. A basal level of phosphorylation at Y418 of Src was observed in control cells. This was significantly enhanced upon stimulation with VPF/VEGF. However, pre-treatment with either dopamine or kinase inhibitor caused a significant decrease in Y418 phosphorylation and, therefore, activation [30] of Src (Figure 3B). Hence, from our data thus far we infer that dopamine by decreasing tyrosine phosphorylation on VEGFR-2 inhibited the association of Src with VEGFR-2 resulting in inhibition of Src activation.

### VPF/VEGF mediated disruption of ZO-1 between the endothelial cells is abrogated by dopamine

**Figure 4:**
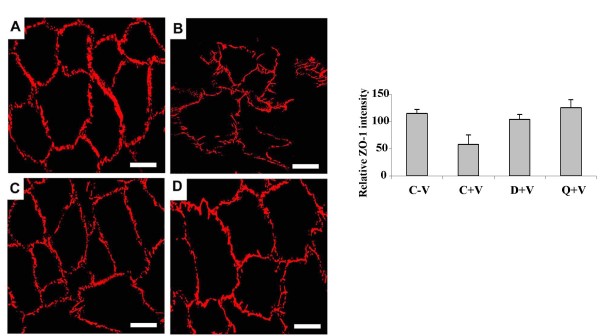
**Confocal microscopy images of tight junctions in HUVEC**. (A) normal ZO-1 in untreated control HUVEC (B) disrupted ZO-1 in VPF/VEGF (10 ng/ml) treated HUVEC (C) dopamine (10 μM) + VPF/VEGF (10 ng/ml) treated HUVEC shows that pre-treatment with dopamine inhibits the ZO-1 disrupting property of VPF/VEGF (D) quinpirole (10 μM) + VPF/VEGF (10 ng/ml) treated HUVEC shows that pre-treatment with quinpirole inhibits the ZO-1 disrupting property of VPF/VEGF. The figures are representative of three separate experiments with similar results. Average intensity of ZO-1 staining for each cell was measured using metamorph software (version 7, Molecular Devices Inc., Toronto, Canada). Scale bar, 20 μm.

Among the tight junctions, ZO-1 plays an important role in maintaining vascular permeability [6,25]. Moreover, recent reports also indicate that the VPF/VEGF induced vascular leak is through the disruption and phosphorylation of ZO-1 between the endothelial cells [6,24-27,31]. We therefore determined whether the action of dopamine is through suppression of VPF/VEGF mediated disruption of ZO-1 in HUVEC. Our confocal microscopy experiments demonstrated that although treatment with VPF/VEGF disrupted ZO-1 between HUVEC in comparison to the untreated controls (Figure 4A and 4B), pre-treatment with either dopamine or quinpirole significantly abolished this effect of VPF/VEGF (Figure 4C and 4D). Neither dopamine nor quinpirole had any direct effects on the integrity of ZO-1 between the endothelial cells (data not shown), thereby confirming that the action of dopamine was VPF/VEGF specific and it was through dopamine D_2 _receptors.

### VPF/VEGF induced association between tight junction proteins, ZO-1 and occludin, are inhibited by dopamine

**Figure 5:**
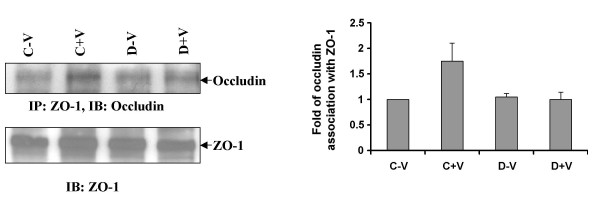
**Effect of dopamine on ZO-1 and occludin association**. Immunoprecipitation experiment showed ZO-1 and occludin were constitutively associated with each other. In the figure C-V is the control, HUVEC without any VPF/VEGF or dopamine treatment, C+V is the HUVEC treated with only VPF/VEGF (10 ng/ml) for 5 min, D-V is the HUVEC treated with 10 μM dopamine for 15 min and D+V is the HUVEC pretreated with 10 μM dopamine for 15 min and then treated with VPF/VEGF (10 ng/ml) for 5 min. The figures are representative of three separate experiments with similar results.

Occludin is an integral membrane protein closely associated with the tight junctions of epithelial and endothelial cells. ZO-1 has been demonstrated to interact with the transmembrane protein occludin, and it has a role in the localization of occludin to tight junctions [32]. Lysates from serum starved HUVEC were immunoprecipitated with ZO-1 antibody and immunoblotted with antibody against occludin. ZO-1 and occludin were constitutively associated with each other. The association between ZO-1 and occludin increased significantly upon treatment with VPF/VEGF (10 ng/ml), and this association was inhibited by dopamine (10 μM) pre-treatment (Figure 5). Our data clearly suggest that dopamine inhibits VPF/VEGF induced association of ZO-1 with occludin and might explain the inhibitory effect of dopamine in VPF/VEGF induced permeability.

**Figure 6:**
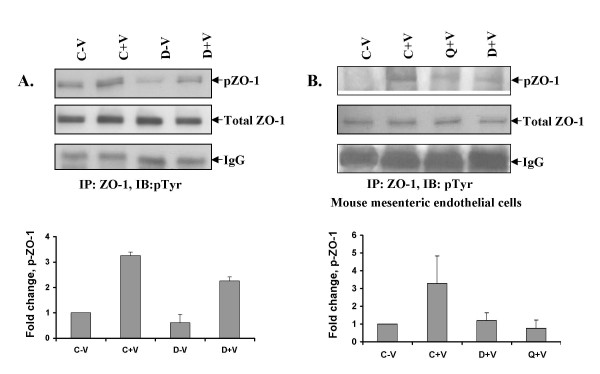
**Effect of dopamine on VPF/VEGF-induced phosphorylation of ZO-1 *in vitro *and *in vivo***. (A) Decreased VPF/VEGF-induced phosphorylation of ZO-1 in HUVEC incubated with dopamine (10 μM) for 15 min before VPF/VEGF (10 ng/ml) treatment. In the figure C-V is control, HUVEC without any VPF/VEGF or dopamine treatment, C+V is the HUVEC treated with only VPF/VEGF (10 ng/ml) for 5 min, D-V is the HUVEC treated with 10 μM dopamine for 15 min and D+V is the HUVEC pretreated with 10 μM dopamine for 15 min and then treated with VPF/VEGF (10 ng/ml) for 5 min. The figures are representative of three separate experiments with similar results. (*B*) Increased VPF/VEGF-induced phosphorylation of ZO-1 in mesenteric vascular endothelium (MVE) of mice treated with 500 ng of VPF/VEGF [C+V] and decreased VPF/VEGF-induced phosphorylation of ZO-1 in MVE of mice treated with either quinpirole (10 mg/kg i.p.) [Q+V] or dopamine (50 mg/kg i.p.) [D+V] 10 min before VPF/VEGF (500 ng) treatment were detected. C-V is the untreated control.

Recent reports indicate that VPF/VEGF induced disruption of ZO-1 in the endothelial cells is associated with increased phosphorylation of ZO-1 in these cells [6,24]. We thus investigated whether treatment with dopamine has any effect on VPF/VEGF induced increase in ZO-1 phosphorylation in HUVEC. VPF/VEGF treatment significantly increased ZO-1 phosphorylation in HUVEC in comparison to untreated controls (Figure 6A). However, cells pretreated with dopamine significantly inhibited VPF/VEGF induced ZO-1 phosphorylation. A similar effect was demonstrated with quinpirole (*data not shown*). Our data suggest that the action of dopamine was through dopamine D_2 _receptors, and we next questioned if this observation could be mimicked *in vivo*.

It was observed that administration of either dopamine or quinpirole into mice prior to VPF/VEGF treatment significantly inhibited ZO-1 phosphorylation (Figure 6B) in the mesenteric vascular endothelium of these animals, thereby validating our *in vitro *results *in vivo*. Overall, our data clearly suggest that VPF/VEGF induced hyperpermeability is controlled by dopamine through its D2R pathways, and it is mainly by controlling posttranslational modification of tight junction proteins.

### Dopamine modulates association between AJ and TJ proteins

**Figure 7:**
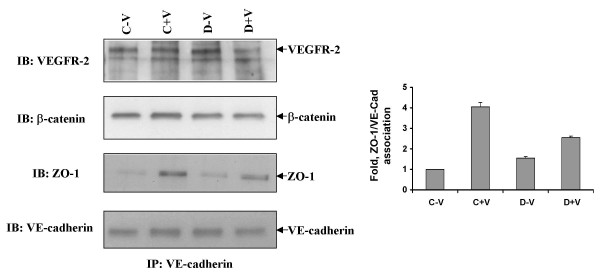
**ZO-1, β-catenin, VE-Cadherin and VEGFR-2 are in the same immunocomplex**. Immunoprecipitation with VE-cadherin antibody showed constitutive association between VE-cadherin, VEGFR-2, β-catenin and ZO-1. In the figure C-V is control, HUVEC without any VPF/VEGF or dopamine treatment, C+V is the HUVEC treated with only VPF/VEGF (10 ng/ml) for 5 min, D-V is the HUVEC treated with 10 μM dopamine for 15 min and D+V is the HUVEC pretreated with 10 μM dopamine for 15 min and then treated with VPF/VEGF (10 ng/ml) for 5 min. The figures are representative of three separate experiments with similar results.

VE-cadherin is specifically localized to the inter-endothelial cell junction [13]. VE-cadherin inside the cell is linked to β-catenin that in turn promotes anchorage to the cytoskeleton. Adherens junction, in particular VE-cadherin, is a target of the signaling pathway that increases vascular permeability through VPF/VEGF [13,33-35]. In addition to influencing the tight junction proteins, signals from growth factors such as VPF/VEGF lead to changes in the phosphorylation of adherens junction proteins like VE-cadherin and associated catenins, thus weakening endothelial cell-cell adhesion and increasing vascular permeability. In order to determine the role of dopamine, if any, in affecting this complex, serum starved HUVEC were pre-treated with or without dopamine before adding VPF/VEGF (10 ng/ml). Then cell lysates were immunoprecipitated with VE-cadherin antibody. Interestingly, we observed that VEGFR-2, ZO-1, and β-catenin were constitutively associated with VE-cadherin (Figure 7). Treatment with VPF/VEGF significantly increased the association between VE-cadherin and ZO-1; however, pre-treatment with dopamine decreased the association (Figure 7). On the other hand, neither VPF/VEGF nor dopamine treatment induced any significant changes in the association between VE-cadherin and β-catenin or VEGFR-2.

**Figure 8:**
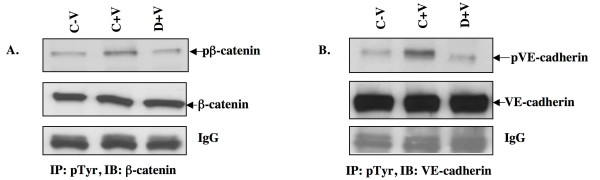
**Dopamine pre-treatment inhibits VPF/VEGF-induced phosphorylation of β-catenin and VE-cadherin**. HUVEC were pretreated with dopamine (10 μM) 15 min before VPF/VEGF (10 ng/ml) treatment. In the figure C-V is control, HUVEC without any VPF/VEGF or dopamine treatment, C+V is the HUVEC treated with only VPF/VEGF (10 ng/ml) for 5 min and D+V is the HUVEC pretreated with 10 μM dopamine for 15 min and then treated with VPF/VEGF (10 ng/ml) for 30 min. The cell extracts were immunoprecipitated (IP) with anti-phosphotyrosine antibodies and immunoblotted (IB) with either (A) anti-β-catenin or (B) anti-VE-cadherin antibodies. The figures are representative of four separate experiments with similar results.

We then investigated whether treatment with dopamine has any effect on VPF/VEGF induced increase in VE-cadherin and β-catenin phosphorylation in vascular endothelium. VPF/VEGF treatment significantly increased VE-cadherin and β-catenin phosphorylation in HUVEC as compared to the untreated controls (Figure 8A and 8B). Even so, dopamine pre-treatment completely inhibited VE-cadherin and β-catenin phosphorylation in VPF/VEGF treated cells. (Figure 8A and 8B).

## Discussion

Here we demonstrate that the neurotransmitter dopamine is a potent regulator of the important signaling cascades controlling VPF/VEGF mediated vascular permeability [3,6], an essential early step in angiogenesis [1,3]. These results provide new mechanistic insights into the dopamine-mediated inhibition of the activities of VPF/VEGF. Src by regulating phosphorylation of key junctional proteins such as VE-cadherin, b-catenin and occludin regulates endothelial permeability (11, 35). Furthermore ZO-1 dissociates from occludin upon phosphorylation by Src (11, 35). Src knockout mouse or otherwise blockade of Src inhibits edema accumulation and thus permeability [28,29]. Here we showed that dopamine inhibited VEGF induced association and activation of Src with VEGFR-2. We also showed that Src Y418 phosphorylation and hence its activation was dependent upon tyrosine phosphorylation of VEGFR-2. Dopamine also inhibited the interaction between TJ proteins and phosphorylation of proteins involved in formation of the AJ complex in endothelial cells. Association of occludin with ZO-1 and ZO-1 with VE-cadherin was significantly inhibited by dopamine in VEGF treated cells. VPF/VEGF induced phosphorylations of VE-cadherin, β-catenin and ZO-1 were also inhibited by dopamine treatment. Various studies have reported that phosphorylation of VE-cadherin or β-catenin correlate with increased endothelial permeability [11,34]. Therefore, we infer that dopamine by decreasing tyrosine phosphorylation of VEGFR-2 inhibits association of Src with VEGFR-2 resulting in inhibition of Src activation. This reduced Src activation then results in decreased phosphorylation of ZO-1, VE-cadherin and β-catenin leading to stabilization of junctional complexes and restoration of endothelial barrier function.

Our *in vitro *results were corroborated by our experiment *in vivo *in that VPF/VEGF induced tyrosine phosphorylation of ZO-1 was significantly inhibited by pre-treatment with dopamine or quinpirole. These results justify our previous report where we showed that mice deficient in peripheral dopamine had increased microvascular permeability following VPF/VEGF administration [21,23]. Our results are in accordance with a recent report that states that hypothermia induced loss of endothelial barrier function can be restored by dopamine pre-treatment [36]. The effect of dopamine thus far is selective for VPF/VEGF, as dopamine does not affect vascular permeability enhancing action of bradykinin or platelet-activating factor [20].

Furthermore endothelial cells expressed D_1_, D_2 _and D_5 _dopamine receptors as determined by western blot and RT-PCR (data not shown); however, the permeability inhibiting functions were mediated mostly by the dopamine D_2 _receptors. In a previous report we had shown that the D_1 _agonist SKF38393 or two other catecholamine vasopressors, epinephrine and norepinephrine, which do not interact with D2 receptors, did not alter or affect tumor ascites [20].

Finally, our result is significant because recent clinical efforts are underway to pharmacologically block VPF/VEGF mediated leakage in patients after acute myocardial infarction [6]. This therapeutic effort in turn may have a major impact on reducing tissue injury and thereby minimize the long-term consequences in this group of patients [6]. A similar approach may become available for stroke patients or individuals with retinal edema, where VPF/VEGF induced enhanced vascular permeability plays an important pathogenic role [6]. Blocking VPF/VEGF mediated vascular leakage can also have a critical effect in cancer patients by reducing metastatic entry of tumor cells into or out of the vascular circulation [1,3,6]. Most importantly, dopamine has been used in the clinics for several years in the treatment of cardiogenic shock complicating myocardial infarction [37]. Taken together, our study suggests a novel and a new therapeutic role for dopamine in these patients. In addition, this report also identifies the molecular mechanism of dopamine's influence on VPF/VEGF induced permeability.

## Conclusion

The main conclusions are: 1. We found VEGFR-2 to be part of a multi-protein complex involving ZO-1, VE-cadherin and β-catenin. 2. VPF/VEGF induced phosphorylations of VE-cadherin, β-catenin and ZO-1 were inhibited by dopamine treatment. 3. Association of occludin with ZO-1 and ZO-1 with VE-cadherin were significantly inhibited by dopamine in VEGF treated cells. 4. Furthermore dopamine inhibited VPF/VEGF induced activation and association of Src with VEGFR-2.

## Methods

### Reagents

ZO-1 antibody (sc-10804), VE-cadherin antibody (sc-6458), β-catenin antibody (sc-7963), Occludin antibody (sc-8144), Src antibody (sc-5266) and VEGFR-2 (sc-504) antibody were from Santa Cruz Biotechnology, California, USA. Anti-phosphotyrosine monoclonal antibody (4G10) and phospho-Src [pTyr^418^] antibody were from Biosource International, Inc., CA; and AlexaFlour 546 secondary antibody was from Molecular Probes, OR. Aclar^® ^fluoropolymer film was from Honeywell, NJ. Dopamine hydrochloride for *in vitro*, quinpirole and all other chemicals if not mentioned were from Sigma-Aldrich, MO. Dopamine hydrochloride for *in vivo *use was from Abbott Laboratories, IL. VPF/VEGF was from R &D Systems, MN. VEGF receptor 2 Kinase inhibitor IV was from Calbiochem (Cat # 676489).

### Animals

Five- to six-week-old NIHBNX-MMC mice were purchased from the Taconic Farms, Germantown, NY. The animals were caged in plastic tubs covered with stainless steel tops at a temperature between 23 and 25°C, humidity level of 50 ± 10%, and a 12:12-h light-dark cycle. The mice were given ad libitum water and food.

### Immunofluorescence

2 × 10^3 ^HUVEC were seeded on collagen coated Lab-Tek chamber slides. After four days of culture when these cells were confluent, they were serum starved for 24 hours. Thereafter, these cells were treated with either dopamine (10 μM) or a specific dopamine D_2 _receptor agonist quinpirole (10 μM) [20], followed 10 min later by addition of 20 ng of VPF/VEGF for 1 h. Thereafter, the cells were washed with PBS and fixed in 4% paraformaldehyde (PFA). Slides were again re-washed with PBS, blocked in 10% goat serum, and stained with anti-ZO-1 antibody (1:100 dilution, except phosphotyrosine antibodies were used at 1:1000) in 1% goat serum for 2 h. Finally, the slides were washed in PBS and incubated 1 hr in AlexaFlour 546 secondary antibody at a dilution of 1:200 followed by postfixing in 4% PFA and mounted in Vectashield (Vector Labs). Confocal microscopy was performed using a Zeiss LSM 510 confocal laser scan microscope with C-Apochromat 63x/NA1.2 water-immersion lense. Absence of signal crossover was established using single-labeled samples [20,38].

### Immunoprecipitation and Western blot analysis in vitro

HUVEC lysates in NP-40 buffer supplemented with protease inhibitor cocktail were immunoprecipitated with different antibodies (1:100 dilutions) and immunocomplexes were captured on protein A-agarose beads. Thereafter, samples were subjected to SDS-PAGE and then transferred to polyvinyl difluoride membranes and immunoblotted. Antibody-reactive bands were finally detected by enzyme-linked chemiluminescence (Amersham) and quantified by laser densitometry [20-23].

### In vitro permeability assay

The assay was performed according to established manufacturer's protocol, Catalog# ECM 640, Chemicon International Inc., CA. Briefly, 2 × 10^5 ^HUVEC in 200 μL were seeded onto collagen coated inserts in 24 well plates. 700 μL complete EBM medium was added to the plates. The cells were allowed to grow for 4–5 days until a monolayer was formed. The medium was replaced with phenol free starving EBM medium containing 0.1% FBS for 12–14 hrs. The respective inserts containing HUVEC were then pre-treated with 10 μM Dopamine or Quinpirole for 15 min. Subsequently, FITC-Dextran containing medium (phenol free EBM) with or without VEGF at 10 ng/ml was added. Fluorescence was measured after 90 min on the Spectra Flour Plus using excitation and emission wavelengths of 485 nm/535 nm.

### Collection of Mesentery from Mice and Immunoprecipitation, Immunoblotting of the mesenteric homogenates

Animal protocols were approved by the Mayo Clinic Institutional Animal Care and Use Committee. Mesenteries were collected from normal 5–6 week-old NIHBNX-MMC mice and treated either with the anti-angiogenic dose of dopamine (50 mg/kg i.p) or specific dopamine D_2 _receptor agonist quinpirole (10 mg/kg ip) 10 min before VPF/VEGF injection. Briefly, normal mice previously treated with either dopamine or D_2 _receptor agonist was injected i.p. with 500 ng of recombinant human VPF/VEGF in 1 ml of HBSS. After 1 h, these animals were anesthetized with an injection of 0.4–0.75 mg/g ip Avertin and were euthanized by decapitation. The abdomen was then opened, loops of the small intestine were exteriorized, and mesentery windows including fatty vascular portions were dissected free and transferred to HBSS at 4°C. Four to six mesenteric windows, each roughly 1–3 cm^2 ^in area, could be isolated from a single mouse.

Thereafter, mesenteries were homogenized immediately upon collection with a polytron tissue homogenizer (model PCU II; Kinematica AG, Switzerland) in a precipitation assay buffer [50 mM Tris (pH 7. 5), 0.25% sodium deoxycholate, 1% NP40, 150 mM NaCl, 1 ml Na_3 _VO_4_, 2 mM EGTA, 1 mM phenylmethylsulfonyl fluoride, 10 ug/ml leupeptin, 0.5% aprotinin, and 2 mM pepstatin A for 10 s at 4°C. Homogenates were then centrifuged for 5 min at 4°C, and supernatants were immunoprecipitated with different antibodies (1:100 dilution), and immunocomplexes was captured on protein A-agarose beads. Finally, samples were subjected to SDS-PAGE and then transferred to polyvinyl difluoride membranes and immunoblotted. Antibody-reactive bands was detected by enzyme-linked chemiluminescence (Amersham) and quantified by laser densitometry [32]. Since these animals were severely immunocompromised (T, NK and B cell deficient), the chance of any immune reaction mediated changes in the vascular permeability (which includes edema) following exogenous VPF/VEGF treatment was negligible.

### Statistics

Differences among groups were evaluated by ANOVA and the unpaired Student's *t*-test or Dunn's multiple-comparison test. Statistical significance was assigned when *P *< 0.05 [20-23]. Error bars were given on the basis of standard deviation (SD) values.

## Abbreviations

VPF: Vascular permeability factor; VEGF: Vascular endothelial growth factor; VEGFR-2: Vascular endothelial growth factor receptor-2; HUVEC: Human umbilical vein endothelial cells; DA: Dopamine; D2DR: Dopamine D2 receptor; VE-cadherin: Vascular endothelial cadherin; ZO-1: Zonula occludens 1; TJ: Tight junctions; AJ: Adherens junctions.

## Competing interests

The authors declare that they have no competing interests.

## Authors' contributions

DM conceived the project. RB participated in the conception of the study, design and coordination and helped to draft the manuscript and performed some experiments with significant help from others as stated. SS performed majority of the experiments and helped to prepare the manuscript. Confocal microscopy was done by SPY. EW and SD performed most of the western blots. CP participated in cell culture and helped with *in vivo *mouse models. All authors read and approved the manuscript.
